# Creativity Is Optimal Novelty and Maximal Positive Affect: A New Definition Based on the Spreading Activation Model

**DOI:** 10.3389/fnins.2021.612379

**Published:** 2021-05-25

**Authors:** Emery Schubert

**Affiliations:** Empirical Musicology Laboratory, School of the Arts and Media, UNSW Sydney, Kensington, NSW, Australia

**Keywords:** creativity, novelty, aesthetic experience, spreading activation, mirroring, pleasure, usefulness, cognitive musicology

## Abstract

Creativity is commonly defined as a process that leads to a novel and useful outcome (an idea, product, or expression). However, two dilemmas about this definition remain unresolved: (1) A strict application of usefulness is difficult to apply to artistic works: who decides what artwork is useful, and how it is useful? (2) The implied boundary conditions of novelty are problematic: The default perspective is that novelty has a monotonic increasing relationship with creativity, or it is categorical—i.e., novel or not. To address these dilemmas, this paper proposes a spreading activation model of creativity (SAMOC), a model built on a brain-architecture-inspired vast interconnected network of nodes, each node representing information, and assigned meanings through interaction with the environment. Nodes are linked to each other according to principles of temporal contiguity (linking objects/events in time) and similarity (linking objects/events by shared features). A node activated by attention spreads through the network through previously linked nodes. Nodes that are well connected activate each other easily, while those that are weakly connected do not. Net total activation corresponds to positive affect (e.g., pleasure), and this is proposed as an essential criteria for a creative work of art, instead of usefulness. SAMOC also predicts that creativity will be optimized at an intermediate, not extreme, level of novelty. Too much activation will occur with the activation of preexisting ideas (hence reproduction rather than creativity), and too much novelty will not produce spread of activation. The two functions (spreading activation and the novelty curve) are superposed to demonstrate this optimal novelty hypothesis. Early evidence of the hypothesis comes from the data that some great works of art were critically rejected at premiers (suggesting excessive novelty), but after sufficient repetition (and therefore linking) became suitably associated and commenced generating activation. The hypothesis has important implications for future empirical research programs on creativity, and for the definition of creativity itself.

## Introduction

Creativity is a process that, from Western perspectives in particular, leads to the production of a novel, useful idea or product (Runco and Jaeger, 2012) and is distinct from reproduction or non-production. It can be broadly conceptualized as consisting of four components: (1) ability (to create), (2) intentionality (to create), (3) a context in which the creativity occurs, and (4) a product is generated that is novel and useful (Walia, 2019; see also Amabile and Pratt, 2016).

Problem solving frequently occupies creativity research investigations. Problem solving that requires a creative solution is quite broad and can be classified as well-defined and as ill-defined (Wu et al., 2017). Well-defined problems have clearly stated goals, such as solving a complicated mathematical equation in a new way or building a bridge over a very long stretch of water. Assessing the usefulness of such tasks is relatively easy, but novelty less so, leading to debate over whether solutions to well-defined problems in fact exhibit overlap between creativity and intelligence, and not exclusively creativity (Kaufmann, 2003; Pétervári et al., 2016). Ill-defined problems require inexact solutions, for example “compose a new piece of music that is 20 min long” or “paint something that moves me,” making them more aligned with conventional conceptions of creativity (Reitman, 1965; Pétervári et al., 2016). As we shall see, defining the process of solving ill-defined problems and assessing them according to the criteria of usefulness and novelty raise questions that are yet to be resolved.

One of the reasons for the impasse stems from the need for testable theory. This paper, therefore, builds on existing models that make explicit, testable predictions about artistic creativity and aim to explain all creativity for the case of ill-defined problems, with the main focus of this paper on the arts, and honing in on examples from music practice and scholarship in particular. Furthermore, rather than building a model around data on creativity, a general model of mental processing is proposed, building on the work of Martindale and Gabora in particular (Martindale, 1995, 1999; Gabora, 2007, 2010, 2016; Vartanian et al., 2003, 2007; DiPaola and Gabora, 2009; Ranjan and Gabora, 2013), which are based on principles of connectionism. This model will be applied to explain data and build hypotheses about creativity.

The paper commences by laying out the connectionist framework from which a spreading activation model is presented. Then, creativity will be modeled, as will aesthetic experience, since the two have important cognitive overlaps that will assist in building a hypothesis. Once these phenomena are modeled, attention will be turned to resolving the dilemmas of novelty and usefulness. The paper then presents evidence for the model and the adequacy of the revised definition.

## Spreading Activation Model

Connectionist frameworks consist of two simple components: nodes and links. “Nodes” encode, store, process, and recall simple pieces of information in a massively interconnected network. The nodes can be referred to as “cognitive units,” mental representations, or schemata, or as the same label as the anatomical source of the analogy—neuron. For the purpose of the present discussion, a simple piece of information will be an object (e.g., a chair, a painting) or an event (a piece of music, dinner), or some component of each. The interconnection of these nodes is achieved by the second component, referred to as a weight or link, analogous to neurophysiological synapses. They link nodes together to different degrees. The linking process takes place through two main mechanisms—temporal contiguity and feature similarity.

**FIGURE 1:**
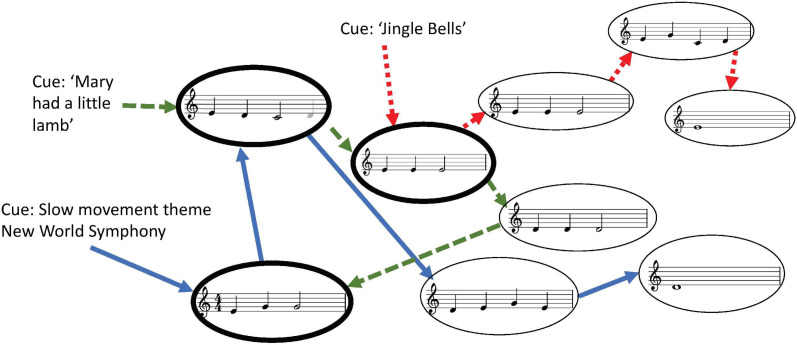
Illustration of veridical chaining, showing music fragments (rhythmically simplified and transposed to C major) parsed into nodes (shown as ovals) and combined through a chaining process. Arrows indicate the chaining links at each temporal step (each color/pattern arrow represents a step in one of the three pieces of music). That is, when the first arrow, cued by the name of the piece of music, activates its target node, the arrow exiting that node primes the next node until the current node has played, and so on. Thick oval nodes indicate those that have been reused in the examples shown.

Temporal contiguity refers to the coding of objects/events in the environment that occur in close succession. Such pairing will lead to the priming (small amount of activation) of the second object/event while the first object/event is the focus of attention. As a simple example, a bar of music in a familiar piece might be represented by one node, which then primes the next bar, and if the next bar is heard, the representation of that bar becomes activated (that is, with additional activation). Figure 1 provides an example of a network where three extracts of music are represented. The sequence of each piece is retained in memory (as indicated by the arrows connecting one bar to the next), but feature similarity means that if a fragment of incoming music is sufficiently similar to an existing fragment (or node—shown as an oval), even if from another piece of music, the node representing that fragment will be recruited, rather than a new node representing the same features duplicating the representation (a process referred to as ‘veridical chaining’ Schubert, 2015; Schubert and Pearce, 2015).

Furthermore, when a node is activated, nodes to which it has been previously paired will themselves be primed (i.e., coming close to activation), or activated, depending on the weighting of the links, and depending on whether other nodes are themselves also activating or priming those nodes. This mechanism of node activation may occur in the form of a transmitter substance (analogous to a neurotransmitter), or as a succession of brief stimulations, referred to as “firing,” where the rate of firing is indicative of strength of transmission. The method of transmission is not of particular concern here as the current application is conceptual rather than biological or mathematical (but for further information, see Smolensky, 1988).

The model presented here necessitates considerable simplification. If we drill down into the node representation of an object/event or part thereof, the node is usually itself interconnected with a “basic feature” node which gives rise to the representation, such as those representing only line angles, color, shape, motion, basic auditory pitch, and so forth. These fundamental building blocks of perception are referred to in some network models of memory as microfeatures (Churchland, 1992; Ranjan and Gabora, 2013) and can also be represented as a more detailed part of the network in the model proposed here but has been omitted for ease of visualizing. While the ensuing discussion treats nodes as representing objects, events, concepts, and so forth, what they reference from the real world need not be fixed for the purpose of the argument being built and will typically be referred to as representations of objects/events or parts thereof, again for convenience and simplicity. Moreover, the extent of activation spread through a network is not determined solely by the weighting of links but also by a concept referred to as temperature (for more details, see Gabora, 2010), where a “high temperature” sets the network up for overall higher connectivity potential—and hence more distant concepts can be more easily activated by than at so-called cold temperature. The principle of network temperature will also be put aside in the present account. Moreover, another simplification is that we will not be considering a special type of link that operates in reverse to the transmission of activation, namely, those “links” that block activation. These “inhibitory links” play an important role in cognition and creativity (Martindale, 1984, 1995; Gabora, 2000, 2016) but will also be put to the side here to facilitate clarity, except to say that they reduce the amount of activation in the network, rather than add to it.

**FIGURE 2:**
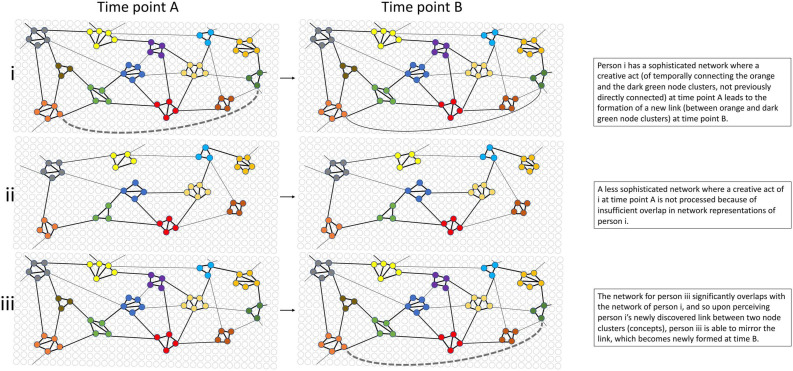
Network representations for three people at two points in time (one time point per column), illustrating the spreading activation model of creativity (SAMOC). It shows clusters of nodes organized into coherent sematic and sequential encoding of objects/events and thoughts, with single colors of closely packed nodes (node clusters) representing a coherent concept, object, or event, and lines between nodes indicating the link strength, with thicker lines indicating stronger links, and therefore a greater propensity to prime or activate an adjacent node, which then spreads through the network according to the weightings of links to adjacent nodes. The dotted line indicates the formation of a new connection between existing node clusters (“transcombination”), either as a result of creative thought or through perception of the newly combined concepts, objects, or events. **(i)** depicts the network for a person who has just made a novel link between two never-before combined ideas (the concepts/objects/events represented by the orange and the dark green node clusters), and so a new link is forming [dotted line in network (iA)]. Over time, the repeated thought or exposure to the new combination strengthens the link between the node clusters (time point B). The new link can be formed through an intermediate node, or directly between the node clusters (the latter shown in the illustration for simplicity). The network for person **(ii)** indicates that this person only shares a small number of concepts that person **(i)** has, and so is unable to process the creative, new link achieved by person **(i)**. At time B person **(ii)** has had no noticeable change in their network, and so will not experience additional activation as a result of being exposed to the newly combined ideas, leading to a non-positive affect experience. Person **(iii)** on the other hand has considerable overlap in mental representations with person **(i)** and so is also able to form a connection between the newly combined clusters (orange and dark green), with the newly forming links appearing at time point B, mirroring that experience by person **(i)** at time point A, and leading to additional activation which generates positive affect (pleasure). The experience for person **(i)** and person **(iii)** is considered creative because previously unlinked nodes have been combined for the first time and generate positive affect. Furthermore, the existing pathways prior to their being combined was relatively distant in terms of the number of nodes that needed to be traversed, and the net, effective link strength. Hence, the greater the separation between nodes (or node clusters) in terms of intermediate nodes and low net link strength, the greater the perception of novelty. There are of course several pathways through which one node can be connected to another distant node in the network, and this is characteristic of the complex dynamic creative system being proposed.

With this spreading activation model, various mental organization phenomena can be illustrated. For example, feather, beak, flight, eyes, and tongue (whether the graphemes, spoken words, images, or multisensory sensation) will each prime a (general) mental representation (or “schema” or “prototype”) of the node representing a bird, as well as activating the nodes representing each of the aforementioned body parts. This collection of related concepts can be illustrated in a network as a number of individual nodes that are strongly linked, using a single color combination of nodes, as in Figure 2 where nodes are shown as small circles in the mental networks of three hypothetical people (persons i, ii, and iii) over two points in time (time A and time B). This form of illustration is based on graph theory, used to understand complex, dynamic, adaptive systems (Gros, 2015). Node representations emerge from exposure to the environment from a theoretically “blank” network, depicted by white circles in the background of Figure 2 (we will mainly focus on the network for person i at time A for now). Wheels, doors, boot, steering wheel, bumper bar, and engine will prime the general mental representation of a car, another group of nodes but of a single, different color in Figure 2 to those represented by the concept of a bird. The two clusters of features are each related to themselves, but distinct from the other (bird and car), and so the links between the concept of a bird and a car are weak. And so in Figure 2, they will occupy two color clusters that are not directly adjacent to one another. However, if a bird and a car are experienced according to one or more of the linking principles, the connections will adjust. For example, the dark blue nodes (in the middle of the illustrated network) may represent the concept of car, and the gray nodes (at the top left) represent the concept of bird. Frequent temporal contiguity of the two concepts can create and increase the direct link strength between the two clusters of nodes (indicated by the line joining the dark blue and gray node clusters in Figure 2iA). As will be explained below, the linking of two *never-before linked* concept or object/event representations (node clusters) is referred to as “transcombination” and is central to the explanation of creativity that will be put forward. This basic architecture will be used to develop a spreading activation model of creativity (SAMOC).

Spreading activation models have been promising in their capacity to replicate human behaviors, such as forgetting and confusing, as well as remembering. Furthermore, a good deal of data support a spreading activation explanation of creativity (Langley and Jones, 1988; Martindale, 1995; Schooler, 1999; Friedman et al., 2003; Sio and Rudowicz, 2007; Cai et al., 2009; Beaty and Silvia, 2012; Gilhooly et al., 2015; Weisberg, 2015; Gilhooly, 2016). Gabora (2010, p. 6) argued that “memory is distributed and content addressable [and this] is critically important for creativity.” By this, Gabora means that memory is not only represented by nodes but that there are overlapping (multiple) pathways to activating nodes, an architecture highly conducive to solving problems in different (including creative) ways—a central advantage of such a mental architecture (see also Gabora, 2002). That is, this mental architecture facilitates “retrieval routes for creatively forging relationships between what is currently experienced and what has been experienced in the past” (Gabora, 2010, p. 6). The spreading activation model applied here is based on the approach proposed by Howes and O’Shea (2014) and incorporates other influences, in particular Martindale (1984) and Thagard and Stewart (2011).

## Creativity as Combination

Creativity researchers predominantly agree that creativity does not take place in a mental vacuum. Even if creative insight may appear to the observer, and even to the creator, as coming out of nowhere, considerable evidence suggests that creativity must involve a combination of existing ideas but, combined in ways that are original and (in the case of some definitions of creativity), solves a problem (Mednick, 1962; Boden, 1994; Baer, 2016). This understanding of the creative mechanism has been discussed in the past in terms of “recombination” (Welch, 1946). Influentially, Boden (2004) proposed two broad forms of creativity that hinge on the combination of existing or newly formed concepts: exploratory and transformational. Exploratory forms of creativity, according to Boden, consist of (re)combination within the same “conceptual space,” such as a creative set of chess moves (from the large but limited set of possibilities bounded by the conceptual space of the rules of chess) or a creative piece of tonal music (bounded by the rules of tonal music, but involving a massive range of possible pitch combinations). Exploratory forms of creativity operate more or less within a single conceptual space. Transformational forms of creativity, on the other hand, take place when novel combinations are made across two or more different conceptual spaces and usually lead to a novel idea that could not have been thought of before. These different forms of representation combinations have inspired mathematical frameworks for implementing creativity in artificial intelligence (Wiggins, 2006). The key point here is that never-before combined nodes are a necessary part of creative processing. This raises a problem of terminology to which we shall briefly turn our attention.

## Transcombination—Combining Never Before Combined Ideas (Nodes)

The term “combination” does not adequately capture the mental process of creativity. Combination, from a mathematical perspective, means reordering items in an array in any way, whereas in creativity combining necessarily refers to the smaller set of reordering that consists only of those possibilities that are *new* combinations. “Recombination” could be construed as putting back pieces as they originally were, hence not creative, but reproduction. Furthermore, exploratory and transformational forms of creativity encompass such a wide domain of possible conceptual nodes that “combination” is both inadequate and non-specific. Therefore, I will use the term “transcombination.”

‘Transcombination’ draws attention to the novelty of combinations that take place within a conceptual space (exploratory form of creativity) but also more aptly describes the transformational form of new combinations. That is, the suffix “*trans*” makes clear that the two ideas being combined have not been combined together in such a way before. For those accustomed to much of the existing literature on creativity, the term has the same meaning as “combination” or as “recombination” (depending on the source) and avoids the need for the clumsy grapheme “(re)combination.”

Another possible term to adopt for this meaning is convolution, as proposed by Thagard and Stewart (2011), but that term has a particular mathematical connotation and specifically refers to an intertwining of mental representations that is proposed as a process distinct from synchronization, rather than a fresh combination of nodes/concepts. The current paper is agnostic about whether “combination” of nodes occur as a result of synchronization (from which comes the adage “neurons that fire together wire together,” after Hebb, 1949) or as a result of convolution. And so the neologism “transcombination” is the catch-all term for the first time a new combination of nodes have been directly linked to each other.

## Unconscious Transcombinations

Because creativity involves intuitive thought processes, meaning that the individual in the act of creating does not need direct conscious access to the creative process [a process frequently referred to as incubation – see Sio and Rudowicz (2007) and Gilhooly (2016)], some views about transcombination are more metaphysical and are not in any obvious way compatible with the combination of existing ideas account. Lavazza and Manzotti (2013) proposed that transcombination alone is insufficient for describing the creative process. The creative act, they argue, must reach outside the set of existing patterns, symbols, and concepts, into an orthogonal dimension that extends existing semantic space. Semantic space, here, can be taken to be an analog of Boden’s concept space. However, Lavazza and Manzotti proposed that this extension is into the environment itself, leaning on William James’ concept of the mental “fringe.” While the finer detail of this argument is beyond the scope of the present undertaking, it is a necessary part of the story because it suggests that transcombination alone is insufficient to explain creativity and that something extra is needed. However, given that this metaphysical treatment of the problem is reliant on conceptual structures that are non-accessible to the individual (Jacoby and Witherspoon, 1982), the additional dimension may still be explicable in the spreading activation model, specifically coming under the transformational form of creativity proposed by Boden, where different conceptual spaces can coexist in cognition. As Simonton put it, “[t]he magic behind the sudden, unexpected, and seemingly unprepared inspiration has now been replaced by the lawful operation of subliminal stimulation and spreading activation.” (Simonton, 2000, p. 152).

The driving principle of transcombination is not that new concepts/ideas are formed but rather that existing concepts/ideas are combined in a novel fashion. An example of this is the artificial intelligence treatment of music composition by Cope (2001, 2006). His experiments in music intelligence are built on the idea that music of a particular style can be broken into its components, separated, and then newly combined to produce original sounding pieces that are still within the style of the original. For example, by parsing Chopin’s piano composition repertoire into small components—let us call the component nodes—they can be recombined (actually, transcombined) within a given musical framework (such as an existing piece by Chopin, but stripped of its musical surface) to produce new sounding works that are stylistically identifiable as Chopin, without the listener being able to detect that old material is being combined (or recycled) in new ways.

The argument by Lavazza and Manzotti (2013), that to be truly creative one must reach out beyond the confines of existing conceptual space, can be dealt with by proposing that the “reaching” may simply take place into existing, but consciously inaccessible nodes. In a discussion of Cope’s EMI system Dennett (2001; see also Mills et al., 2018) referred to combination (here, transcombination) as being comparable to walking into a messy room and discovering things that trigger a new solution to a problem. The messy room, in the present framework, is a collection of nodes that exists below consciousness but are accessible during periods of (possibly unconscious) creative incubation (for a more recent explanation of creative idea generation that is also highly compatible with the spreading activation account, see Gabora, 2016). And so even if one must seek inspiration from outside the consciously accessible nodes, transcombination can, and probably does, still come from existing mental representations.

In the SAMOC spreading activation model, consider, then, a number of distinct, weakly linked networks of nodes, each node representing a range of previously associated objects/events (see, for example, the visual representation in Figure 2iA as discussed above). During the creative process where a problem requires a novel solution, the solution is achieved by an “intersection of paths of spreading activation” (Schooler, 1999, p. 353) from these previously weakly or unlinked node clusters. To illustrate this, in Figure 2iA a cluster of nodes (circles) with the same color have been previously linked because they represent a reasonably coherent concept. That is, the link strength is generally stronger between nodes within a particular (single colored) cluster than are links across adjacent clusters of nodes. However, note, too, that most of the (like colored) clusters can indirectly be connected to any other cluster. However, the likelihood of this occurring diminishes based on the number of intermediate nodes that separate them, and the net link strength of that pathway (keeping in mind that there are alternate pathways in this complex, dynamic system). In the case of Cope’s experiments in musical intelligence, each of the Chopin fragments can be viewed as occupying a node cluster in Figure 2iA and that non adjacent clusters were being transcombined to create a satisfying, apparently novel Chopin composition.

## Examples from Western Music History

In Western art music composition from around 1650 to 1800, theory was built on the idea that musical harmonic progressions should move from tension to release, with sophisticated rules of harmony and voice leading driving how to set up a harmonic dissonance and how that dissonance should then be resolved (Rameau, 1722/1971; Aldwell, 1989). Variants of these rules would appear throughout this period. These variants are exploratory forms of creativity. However, the general idea that a harmony did not need to be resolved according to the established tension-release principles in Western art music did not occur, in general (although there were exceptions), to composers until the mid nineteenth century when Eastern ways of thinking started to bear influence on philosophers such as Schopenhauer, and in composers such as Wagner in particular, who was himself influenced by Schopenhauer (Wagner, 1987, p. 323). That is, combining Schopenhauer’s ideas with resolution of harmony produced a translational form of creative transcombination. Wagner’s idea of delaying the resolution of dissonance was revolutionary. Another example was the translational extension of this idea in the early twentieth century, with the rejection of the tension and release script altogether, replaced by the “emancipation of dissonance” (Schoenberg, 1950, p. 48) in music compositions. This change culminated in dodecaphonic (12 tone) technique of the second Viennese school, where instead of following the rules of tension release where particular notes were favored over others, all 12 tones of the scale would be treated as equal, which to the ears of people lacking familiarity with the system would mostly sound like dissonances moving to dissonances, tension to tension, a disturbing, translational development in musical ideas, a development that is doubtless as controversial as it is creative—a point to which we shall return.

As another example, John Cage’s reading of Eastern philosophy, which informed his interest in removing determinism and ego from music (Kahn, 1997, p. 559), led to the unlikely (again, transformational) transcombination of ideas of a piece of music consisting of a musician sitting at a musical instrument and remaining silent for the entire performance, as was the case for the piece titled 4′33″. The composer and the performer had their ego removed from the musical process and allowed the “music” to “just be itself” by being the sounds in the environment. Cage reflected on his compositional approaches—“I do not wish blamed on Zen, though without my engagement with Zen […] I doubt whether I would have done what I have done.” (Cage, 1961/2011, p. xxxi). The idea of a delayed and then abandoned resolution was once again pushed forward with the idea of music as (unobtainable) silence, ideas that are still quite surprising to those immersed in the tonal music traditions of per-twentieth century Western Art music and in popular music of the 20th and 21st centuries. However, a close examination of these creative processes can be traced to a novel combination of existing ideas. Furthermore, these transcombinations can occur in many ways and at many levels. As another example, in jazz forms Gatherer (1997) suggested that particular styles of music emerged from new combinations of preexisting styles, such as Bebop emerging from a transcombination of Swing and Blues-oriented Jump style (p. 78).

However, how do we know if these new works are creative? Novelty can be explained by the inverse of link strength between node clusters. Usefulness is more difficult to explain. To understand the dilemma of usefulness as a criterion of artistic creativity, we need to examine the spreading activation model as it applies to the *perception* of an artistic work, namely, “aesthetic experience.”

## Aesthetic Experience

Aesthetic experience in Western cultures is concerned with the reception of a created product and involves contemplation of, or engagement with, that product. A conventional definition of aesthetic experience is that it is the contemplation that results from engaging with an object or event of beauty (Santayana, 1896/1955; Bundgaard, 2015). However, aesthetic experience can be defined more generally as one that includes a significant positive affect (such as feeling awe or being moved) as a result of contemplation of or engagement with an object or event (Schubert et al., 2016). The present discussion is limited to the perception of art works and excludes aesthetic experiences that occur in response to objects/events that serve a purely practical function and those found in nature. Because the focus of the current investigation is on creativity, we will focus on the special case of the artistic output: when a new work is experienced for the first time. After all, this is when an individual is likely to be struck by the creativity of the new work.

## Aesthetic Experience as Transcombination and Mirroring

As discussed in the introduction, veridical chaining is compatible with the spreading activation model. As a fragment of music is heard, a mental representation (node) that matches the unfolding music is activated, and this activated node primes the representation of the next part of the music that has been encoded earlier (Figure 1). Hence, familiar music will activate a temporally measured cascade of mental representations, with each mental representation being primed and then activated in succession, synchronized with the unfolding music. With each listening of the familiar pieces, the link strength between those nodes increases. This corresponds to the experience of increasing familiarity. The increasing link strength continues with additional exposure, unless the exposure is high in frequency and massed in which case habituation will occur (discussed below).

However, if a new piece is heard, there may not be as many nodes to activate, or different nodes to the ones that are primed are activated (referred to as disruption of expectation in the influential work of Meyer, 1956). In this case, we may be experiencing something new or novel. This is illustrated in Figures 2i,iii, noting the flexibility of the network representation—in the earlier example, the same illustration was used to represent concepts of physical objects (birds and cars), and here it represents music. Upon listening to a new creative work, created by the individual represented in Figure 2iA, if the person indicated in Figure 2iii initially (that is, at time point A) had the nodes representing the components of music which the person represented by Figure 2iii has transcombined, then repeated listenings by the person of Figure 2iii will strengthen the links between those through temporal contiguity (Figure 2iiiB). Thus in the example, two weakly linked node clusters are activated by the new piece of music, and the initially weak link strength between them is altered, with links strengthening (in the Figure, the dotted line between the two nodes indicates the new formation of a link, which by time point B of Figure 2iB has become an established link). The network of person iii, through the perception of person i’s created work, comes to mirror the network of person i.

However, for the network of the person represented in Figure 2ii, a part of the music is not represented at all (the dark green node cluster is missing because it has never been formed), and so no new activation can take place. The new piece may be assessed as incomprehensible to that person. Similarly, if the two nodes representing the new idea do exist, but are too weakly connected, activation induced by the new music may simply not be sufficient to create or strengthen a link through the intermediating nodes of the network. That is, the stronger the links between node clusters, whether through an alternate pathway via other nodes, or directly between the two, the easier it will be to transcombine them. However, if the two clusters are very strongly linked then the link between the concepts is already represented (familiar), and while potentially generating activation, they do not satisfy the condition of being sufficiently novel to be judged creative. We must therefore now take a closer look at how novelty and usefulness are modeled by SAMOC.

## The Dilemma of Novelty in Creativity

Novelty is one of the two common criteria listed when defining creativity (Runco and Jaeger, 2012). An inherent problem is what level of novelty is necessary to constitute an assessment of creativity. Must a particular level of novelty be surpassed (meaning that novelty is categorical), or is there a monotonically positive relationship, where provided other criteria of creativity are met, increases in novelty translate to increases in creativity? Styhre (2006, fn. 1, p. 148) went as far as to suggest that novelty is not central to creativity but rather “connectivity, associations, assemblages and multiplicities point at the combinatory nature of creativity,” which is highly compatible with the spreading activation model. Diedrich et al. (2015), on the other hand, evidence the key role novelty plays in creativity.

**FIGURE 3:**
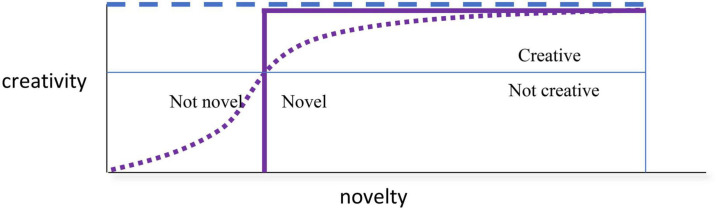
Hypothetical creativity assessment as a function of novelty. Thick solid purple line indicates simple threshold approach to assessing creativity. If novelty exceeds the threshold, the novelty criterion is satisfied. The monotonic increasing function is illustrated by the dotted curve. The functions are adjusted in this illustration such that they produce an assessment of “creative” (i.e., above the thin blue horizontal line) at the same threshold level of novelty. The dashed line is a reminder that the “usefulness” criterion has been met for all levels of novelty shown.

In his discussion of the matter, Kaufmann (2003) reported diverse views in the literature ranging from those who define just “different” as sufficiently meeting the novelty criterion, through to those who apply complicated, crude, or non-distinguishing conceptions of novelty. Treating novelty as an above-threshold category (an object/event is novel or it is not) fails to recognize the existence of degrees of novelty. One solution is to consider the category as a simplification of something that actually varies in concert with creativity itself—more novel is more creative. Figure 3 illustrates the way that the novelty criterion is interpreted according to the threshold (filled line plot) and monotonic increasing (dotted curve) methods, these being the easiest to pin down to a simple conceptualisation of novelty. As we shall see next, this pattern is in conflict with (or opposes) another criterion of creativity.

## The Dilemma of Usefulness in Creativity

Usefulness is the other one of the two basic, and most frequently reported, criteria of creativity (Runco and Jaeger, 2012). The process of creatively solving well-defined problems is highly amendable to assessment in terms of usefulness. If the problem is legitimately solved, the solution is useful. However, more disputed is whether usefulness is a concept that can be aptly applied to creativity in artistic endeavors, where problems are generally ill-defined. Answering the question “how is a song or a symphony useful” could raise a wide range of rather subjective responses, making empirical investigation problematic, and suggests that such a criterion misses the point. Some prefer to apply, instead, the criterion of “value,” because it better reflects the personal nature of aesthetic experience of art as judged by the perceiver. Valuing a work of art is a more plausible way of referring to the concept that one is trying to capture when assessing the “usefulness” of a work of art. However, even this term is problematic because value is also to some extent subjective (Weisberg, 2015).

In a critique of creativity definitions, Harrington (2018) suggested reconsideration of an older term, still in use today, of “satisfaction” (Stein, 1953), rather than usefulness or value. Satisfaction is a term that appears to capture aspects of usefulness and value as applied to the arts and so lends itself to the definition of creativity. Satisfaction also has the advantage of providing a tangible, potentially reliable understanding of the aspect of usefulness relevant to assessing creativity, but it does not capture the richness of the creative and aesthetic artistic experience.

Given the problematic nature of the usefulness criterion, it is worth considering an alternative aspect of creativity that until recently has received less attention but broadens out the limited satisfaction option: the affective component of creativity. There is a growing body of evidence that the mental processing and outcomes of creativity generate positive affect (Russ, 1999; Henderson, 2004; Amabile et al., 2005; Bledow et al., 2013; Tavares, 2016; Gu et al., 2018). Positive affect is a broader concept than satisfaction, incorporating experiences reported as a result of the creative process (such as “aha,” “wow,” surprise, …) (Wiggins, 2006; Macedo et al., 2009; Thagard and Stewart, 2011; Becattini et al., 2017) as well as the reception of the artistic output (e.g., awe, being moved, thrills) (Konečni, 2005; Schubert, 2009-2010; Schubert et al., 2016). Fortunately, there is a straightforward theory about the underlying mental mechanism of positive affect that has been applied to the spreading activation model.

## Positive Affect as Spreading Activation

Martindale proposed a simple relationship between the net amount of spreading activation and the amount of pleasure experienced—suggesting that they have a monotonic increasing relationship. More activation is experienced as greater pleasure—the “pleasure of thought” principle (Martindale, 1984, for a similar, more recent perspective, see Hofmann and Jacobs, 2014). Despite building this finding around a considerable battery of evidence (for a summary, see Martindale and Moore, 1988), Martindale’s ideas have been criticized, above all for the simplistic characterization of aesthetic experiences of beauty, which he was accused of reducing to mere preference (Croft, 2011), a criticism that has its parallel in the criterion of satisfaction applied to creativity, discussed above.

However, this concern has been reconciled in recent years with the idea that preference falls under a broader category of experience labeled “positive affect valence” (or, in the present discussion, “positive affect”) (Colombetti, 2005; Schubert, 2013). Schubert et al. (2016) proposed that affect valence could be divided along a number of dimensions—most pertinently here as valence (positive or negative) and hedonic (shallow and deep) tone. Positive affect is a feeling that is contemplative but also drives an individual to repeat, continue, or seek out the future activity that leads to that feeling. It can be thought of as attraction in the broadest sense. Negative affect is a feeling related broadly to aversion, repulsion, aggression, or boredom that usually drives the individual away from the activity that leads to that feeling. Positive affect defines aesthetic experience according to some researchers (see Schubert et al., 2016).

Within positive affect, there are different levels of depth of experience. Empirical aesthetics researchers frequently measure shallow hedonic tone (such as preference, enjoyment, liking) because these are easy to collect and quantify via self-report, and reasonably reliable (e.g., Hardiman and Zernich, 1975). Deep hedonic tone, on the other hand (for example, feelings of awe, being moved, etc.), is considered more reflective of fully fledged aesthetic experience but occurs less frequently (Konečni, 2005). Shallow hedonic tone, when positive (e.g., liking), can therefore be taken to be an index of the broader, richer concept of positive affect, and aesthetic experiences in general, even if it may miss the essence of many aesthetic experiences.

An alternative reason for attraction to art is that engaging with art triggers neuroanatomically localized pleasure centers. The pleasure of thought principle, however, is not dependent on anatomical centers, because pleasure, and positive affect in general, is generated by the process of distributed activation, not the stimulation of a particular, specialized center. While the proposed model does not rely on neuroanatomical evidence (it is a cognitive, conceptual model), it is interesting to note the growing number of studies that refer to networks, circuits, and distributed anatomical structures that are active during periods of pleasure, moving away from specialized center-based locations. For example, Berridge and Kringelbach explain, “wanting for rewards is generated by a large and distributed brain system. Liking, or pleasure itself, is generated by a smaller set of hedonic hotspots within limbic circuitry.” (Berridge and Kringelbach, 2015, p. 646; see also Knapp and Kornetsky, 2009; Schubert, 2012; Lesage and Stein, 2016).

## Positive Affect From the Perception of Familiar Art

For the spreading activation model, if activation leads to positive affect (not just preference), perceiving the familiar should produce strong positive response. Activation of a node representing a portion of music that is familiar means that the next, incoming portion of music will activate the node that was already primed, leading to an increase in net activation (Figure 1). Evidence supporting this claim is abundant—people like stimuli, and works of art in particular to which they have been previously exposed (Zajonc, 1968, 2001; Finnäs, 1989; Cutting, 2006; Schubert et al., 2014; Chmiel and Schubert, 2017).

However, this leaves us with a puzzle: people should gravitate toward experiencing familiar works of art and never to any new works. New combinations of music representing nodes would not generate as much activation as familiar combinations. Why would anyone want to experience something new and creative according to the SAMOC spreading activation account? There are several reasons, but we shall focus on one for now: that by experiencing only the familiar, habituation will take place. The discussion of habituation that follows turns attention back to creative production, rather than perception (of creative works). However, the principles of pleasure (positive affect) induction from perception described above remain the same as for creative production, because the mental processing takes place in the same network architecture and principles (hence they are mirrored). The shared framework is based Martindale’s connectionist approach in which he recognized that “[t]he act of creation, a case of extremely successful cognition, is […] isomorphic with the perception of something of great beauty” and that “the act of creation and the perception of beauty are essentially identical” (Martindale, 2001, p. 25 and p. 33).

## Habituation

When a node is activated frequently (through massed repetition), the node fatigues and its capacity to transmit to other nodes decline (e.g., due to a decline in its firing rate), ceasing to propagate activation, and itself failing to contribute to the activation. As a result, the individual will stop showing interest in the overexposed stimulus, possibly experiencing negative affect such as boredom (Schubert et al., 2016). The individual can seek other ways of increasing activation. One way to do this is to engage with stimuli that have existing mental representations but have not recently been activated. This gives rise to the finding that artistic stimuli remain much loved over a long period of time (Martindale et al., 1990). However, the revisiting of stimuli after a long absence would not in any obvious way produce a sense that the work was creative, even though its re-perception may well generate considerable pleasure.

Another strategy, of interest here, is to combine existing mental representations in new ways. By forming new combinations between weakly linked nodes, net activation in the network can under some circumstances once again be increased. If one has habituated to a stimulus, then finding another stimulus that provides more activation should not be difficult. The question then becomes, what combination of mental representations would optimize this activation? And pertinent to the current discussion—which combination would also be considered creative? Figure 2 helps to answer this question. Let us suppose that two adjacent clusters of nodes (consider two node clusters represented as two different colors in the Figure) that are closest to one another are no longer activating due to fatigue. More activation can be generated by turning attention to the links that are weaker, but not fatigued, with a displaced (rather than adjacent) cluster. In the case of the creative endeavor, the creator seeks out the pairs of node clusters that are as far apart as possible, while still containing some (albeit weak) links. Combining these is satisfying (positive affect) because activation is recovered, and also the link strength is increasing between these, until now weakly combined nodes, through the formation of a new, but more direct link (see the dotted line in Figures 2iA,iiiB). That is, link strength increases if the transcombination is repeated, or if the two ideas are already sufficiently linked.

In the case of music, this cognitive explanation can be used to model any of the forms of musical delivery discussed above—composition, improvisation, performance, and imagination. Examples of compositional creativity were discussed above. In the case of improvisation, it might be the surprising combination of different ideas (see also Spence, 2021). In classical performance, it might be the satisfying outcome of applying a historically informed interpretation to a piece of music that had not had such treatment. Each of these examples can be explained in terms of transcombination of nodes, and each will be satisfying (i.e., produce positive affect) because existing mental representations have been activated where one or more of the mental representations have not been fatigued, meaning that greater activation is possible than before the creative thought came to mind.

The assumption of this kind of creativity is that mental representations of (in this case) music exist and should not have been previously combined, and that at least one of the nodes being stimulated are not fatigued. The existence of mental representations sets a boundary in terms of what can be considered creative. Novelty alone cannot explain this criterion because novelty alone could mean absence of mental representation, or mental representations that are completely unlinkable to one another. The current model would not produce increasing activation if a link was being made between an existing node and one that does not as yet exist. This is simply not possible. The individual could not process the new object/event without additional experience. The perception of such a combination would be incomprehensible.

## Optimal Novelty Hypothesis

To optimize activation using novel combinations (as distinct from those that are already well linked), one does *not* select only frequently combined mental representations. Those representations must be inhibited during creative processing to avoid reproduction/replication—by definition not creative, as discussed in Section “Spreading activation model.” However, similarly, highly unrelated representations should not be combined because they do not have sufficient, existing link strength, which translates phenomenologically to being too incongruous (too translational, if you like). The rationale for this assertion requires some explanation. Imagine, for example, a highly creative individual, who also has a wide range of experiences (Figure 2i). This individual has a large number of mental representations, and the individual has the capacity to combine quite diverse nodes together. Transcombining nodes that are quite conceptually remote will not be as difficult for that person as it would be for someone with less aptitude for creativity and fewer experiences (Figure 2ii). Like the regression to the mean problem (Bland and Altman, 1994), the creative individual seeking to appeal to a wide audience rapidly (if that is what she/he is aiming to do)—such as at a premier public presentation (performance, exhibition etc.)—will be better off not selecting the most diverse mental combinations that satisfies perhaps only their own mental network (and hence their own creative goals). Some calibration in the level of novelty of the combination is required to increase the appeal of the creation to a wider cohort. Whether considered too outlandish for the creator, or thought to be too difficult for the target population to process, such unusual combinations, too, will be inhibited. In this case, the creator would not wish to produce something that is new and thrilling (high positive affect) to them because it will miss the mark for the more typical audience. The aim of the creator will be to reach the limits of combinations that the most typical recipient is likely to experience.

**FIGURE 4:**
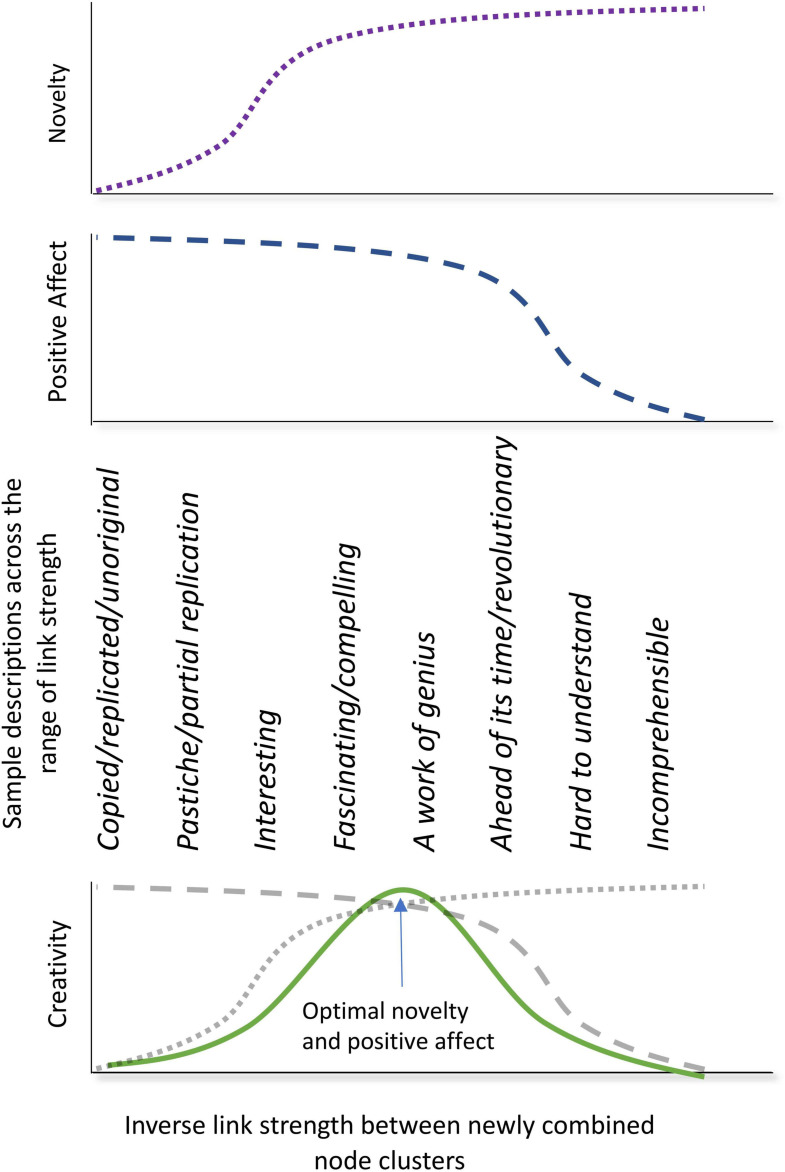
Demonstration of the optimal novelty hypothesis—why novelty must be at an intermediate level rather than monotonic increasing to maximise creativity. The top curve indicates the implicit alternative (conventional) view that novelty increases monotonically as a function of decreasing link strength between transcombined node clusters. That is, more conceptually distant node clusters have a weaker link strength than node clusters that are conceptually more related. Below this is a plot of the activation strength decreasing as link strength decreases. Here activation is related to positive affect, and so we see less positive affect as more novelty is experienced. The bottom, green curve shows the schematic superposition of these two curves, which gives rise to a maximal level of creativity, where creativity is defined in terms of optimal novelty and positive affect. Note that positive affect is proposed as a better indicator than “usefulness” when assessing creative works, as explained in the body text.

An inverted-U of positive affect as a function of novelty is therefore the way to think of producing a creative work. The (possibly unconscious) aim of the creator, in this situation, is to produce node combinations that will maximize the activation of the perceiver. If the creator has no systematic way to directly access this information they would need to do it through intuition, and this may also involve researching their prospective audience. This optimization of the two systems—novelty and positive affect—is illustrated in terms of SAMOC in Figure 4 and constitutes the optimal novelty hypothesis.

The reasoning for the creator taking control of what nodes to make available for recombination is similar to that used by Martindale, after Eysenck, which hints at the idea of an optimal amount of novelty:

*if we let nodes come on and off totally at random, the “search space” for problem solving of other than a trivial nature is so large that a solution could never be found. We must cut down what he [Eysenck] calls the “associative horizon” to a reasonable level. If we trim it too far, though, only “relevant” nodes will be activated, and they do not contain the crucial hint. We want a network that will at least periodically go to a low-arousal state in order to “search” for a solution and return to a higher arousal state [activation] to see if the solution is a good one (Martindale, 1995, p. 261).*

## Evidence of Non-Extreme Novelty From Historical Reception of Compositions

Evidence for optimal rather than extreme novelty can be found in the catalog of critically derided first performances of pieces of music that would later become important works of art (Slonimsky, 2000). A striking example is the reception of Beethoven’s Ninth Symphony, now treasured as an iconic work in the music repertoire. Early performances were greeted with at times considerable taunts, including outright rejection of some of the symphony’s most innovative features—the size of the orchestra, and the final, choral movement, which attracted commentary from William Ayrton about the “acoustic missile” of instruments that

*… made even the ground shake under us, and would, with their fearful uproar, have been sufficiently penetrating to call up from their peaceful graves … the revered shades of Tallis, Pursell, and Gibbons, and even of Handel and Mozart, to witness and deplore the obstreperous roarings of the modern frenzy in their art. (cited by Cook, 1993, p. 42).*

and, regarding the innovation of a choral movement, Ayrton remarked

*[w]hat relation it bears to the symphony we could not make out; and here, as well as in other parts, the want of intelligible design is too apparent. In quitting the present subject, we must express our hope that this new work of the great Beethoven may be put into a produceable form; that the repetitions may be omitted, and ***the chorus removed altogether***; the symphony will then be heard with unmixed pleasure, and the reputation of its author will, if possible, be further augmented. (cited by Cook, 1993, p. 43) [emphasis added].*

Beethoven’s transcombination of choral music and symphony was not mirrored by this critic.

There are documented cases when Beethoven deliberately chose weakly linked nodes to transcombine, reflected in his disdain for audiences who found his music incomprehensible. Novelist and critic Ludwig Rellstab was one of those who was perplexed by Beethoven’s later works, but when speaking with Beethoven recounted to the composer a performance of the Op. 127 quartet, stating: “‘It had been carefully practiced and was played twice in immediate succession’. Beethoven’s reply was: ‘That is well. It must be heard several times”’ (Adelson, 1998, p. 236). Beethoven’s presumed reply is realized in the spreading activation model as different musical material being too unrelated for some audience members, and so needing further exposure to increase the link strength between the new musical ideas, or, if necessary, to form the musical ideas that are not yet represented.

For highly creative, highly experienced individuals, their positive affect function of novelty (Figure 4) will be broader than that of the typical person, and unless they are willing to wait for future acceptance or even recognition of their creativity (Beethoven, Wagner, and Schoenberg had each indicated that they were writing music for the future—see, e.g., Hueffer, 1874), they would need to produce works that made less novel combinations for them than they may typically do. Beethoven did with his “Battle Symphony,” Wellington’s Victory, which quoted familiar patriotic tunes and applied real-life military sound effects—all familiar to the large audiences that adored the work, but the piece would come, in time, to be considered an embarrassment by later connoisseurs of Beethoven’s music (Cook, 2003). Furthermore, composers also made changes to their compositions if the pieces were received poorly (Anderson, 2017). Each of these cases points to a calibration that composers may or may not make to their new compositions for audience accessibility, while remaining creative, or that some form of repetition is needed so that the audience can process the newly connected ideas, and, from the SAMOC perspective, increase the link strength between them, hence increasing positive affect.

## Evidence of Transcombination From a Music Analysis Perspective

There is abundant evidence of the creative process in music composition consisting of modifications or adaptations of preexisting musical fragments and, unless explicitly quoted, appearing in the new composition as a result of unconscious processing. The creator need not have conscious awareness of the transcombination of existing musical ideas, such as musical fragments (Figure 1) and templates (formal structure, beat pattern, harmonic progression, etc.). Cope refers to these as musical allusions, and Jan, building on Cope’s groundwork, as memes. For Jan “any discrete musical segment which a composer assimilates from his or her cultural environment may be regarded as memetic” (Jan, 2007, p. 60). The important point here is that the allusions or memes are generally fragmentary and that a single meme cannot on its own constitute a new work, unless the new work is intentionally presented as a tribute or quotation (or more euphemistically, “borrowing.” see also Burkholder, 1994). That is, if a meme is too long (temporally, or structurally), or the similarity to its source is too salient, the assessment of the work as a new creation is inhibited.

Furthermore, transcombination and exploratory (as distinct from transformational) forms of creativity suggest that new composition will consist of stitching together of different memes. Jan (2015) presents sophisticated accounts of how this works based on his bio-evolutionarily inspired analytic technique. In one analysis, he presented a score of a phrase from Beethoven’s Piano Sonata op. 110, Movement I, and mapped out the sources of the identifiable memes. Within the space of a few bars, links were made to works by Haydn, Mozart, J. S. Bach, and Beethoven’s own earlier outputs. Jan was cautious to point out the complexity of performing such an analysis, with two critical issues worth mentioning here. First, a musical meme transmits through several pieces of music, from person to person, and generation to generation. To select a single source of the meme simply indicates that it has been in existence, rather than meaning that the principle, single source of origin has been located. Second, performing such an analysis is extremely difficult without extensive knowledge of music. Jan (2004) points out that the vast amounts of information that needs to be parsed by the researcher requires computation tools, which are far from fully developed. That is to say, if one fails to parse a piece of music into its component memes, it could mean that the transcombination explanation is unsupported, but it could also mean that the memes have yet to be located. The present paper hinges on the latter case prevailing.

## Conclusion

This paper proposed a spreading activation model to generate theory about creativity—SAMOC. As a result, the conventional definition of creativity—that it is the process leading to useful and novel outcomes—came into question. By viewing ideas as nodes that are part of a massively interconnected network operating under reasonably simple principles, a proposal for an alternate definition of creativity presented.

In everyday language, creativity is the process that leads to an outcome that generates positive affect (e.g., pleasure) and is optimally (not extremely) novel. In terms of the spreading activation model from which these conclusions were drawn, and in more rigorous language, creativity is the process of transcombining (forming new combinations of existing) nodes such that they produce a large amount of net activation, even though they will have progressively weaker links between one or more pairs of the nodes in question as novelty increases. In brief, the creative work can be defined as a work which is sufficiently novel to produce positive affect. This is in contrast to conventional definitions that focus on usefulness and novelty. Usefulness cannot be a criterion of a work of art unless the usefulness is mediated by some other criterion. That criterion is the aesthetic experience, which is the positive affect that occurs due to the contemplation of or engagement with (usually) a work of art.

Creativity by one person and the perception of creativity by another person are intricately related through cognitive organization. Based on the above argument, the relationship can be thought of as the successful mirroring of the transcombinations of existing nodes by the perceiver of the creation. The creator and perceiver must each share node representations, and the nodes which have been newly combined by the creator must also be newly combined by the perceiver. The creator chose that combination of nodes because it produced a significant amount of spreading activation, and therefore positive affect. The perceiver, in the process of transcombining the corresponding nodes, also experiences positive affect, and this is caused, according to the present account, by the additional spread of activation that the newly formed connection generates.

While the focus of the present investigation was on ill-defined problems—namely, the creation of artistic works—the newly proposed criteria for creativity may apply to creative solutions to well-defined problems. It seems likely that usefulness will correlate highly with positive affect. Evidence is emerging of the positive affect that is associated with the creativity of new outputs and the reception of those outputs (Russ, 1999; Henderson, 2004; Amabile et al., 2005; Wiggins, 2006; Macedo et al., 2009; Thagard and Stewart, 2011; Bledow et al., 2013; Tavares, 2016; Becattini et al., 2017; Gu et al., 2018). The question for future research is whether this positive affect is a substitute for usefulness, an enhancement of usefulness, a completely separate criterion, or simply a by-product of creativity. The optimal novelty hypothesis, too, may apply to creative solutions for well-defined problems. Although novelty is not such an important criterion for well-defined problems, according to the optimal novelty hypothesis, if a well-defined problem has several solutions, the most creative solution will be the one with an intermediate, rather than a very high amount of novelty.

The proposed model and the predictions it makes are testable. The evidence reported in this paper focused on historical documents about Western music composers, and examination of an innovative meme identification analytic technique of the musical record. However, the theory also makes empirically testable predictions, particularly with regard to degree of novelty that is required to procure a judgment of creativity. For example, a carefully designed study could investigate creativity ratings of three artistic outputs that exhibit different levels of novelty. The optimal novelty hypothesis states that the most creative output will be the one that is slightly less novel than the most extremely novel output, but more novel than the least novel output, when all other factors are held constant. That is, the optimal novelty hypothesis is falsifiable, and the historical evidence reported lends support, but more controlled testing is needed.

The model is also compatible with the data presented by Diedrich et al. (2015) that argues for the primacy of novelty in determining creativity, even for products initiated through ill-defined (artistic) criteria. The primacy argument holds that if and only if the product is novel might it be considered creative. That is, by arguing that an intermediate amount of novelty optimizes creativity, the critical role of novelty in creativity is not at all diminished. Furthermore, the model can be applied to different categories of emotion. Even though the evidence presented in this paper is based on the “Big-C” creativity because it drew data from culturally eminent musicians, the network architecture employed still operates on the same principles for non-eminent categories of creativity that are concerned with everyday and developmental aspects of creativity (Beghetto and Kaufman, 2007; Schubert, 2012).

The SAMOC spreading activation model provides a framework for understanding creativity and has helped to interpret findings from a theoretical perspective. Having the theoretical framework that builds on past conceptualizations of mental processing, memory, and creativity will help to provide structure and direction for future research programs in creativity as well as other human behavioral pursuits.

## Data Availability Statement

The original contributions presented in the study are included in the article/supplementary material, further inquiries can be directed to the corresponding author/s.

## Author Contributions

The author confirms being the sole contributor of this work and has approved it for publication.

## Conflict of Interest

The author declares that the research was conducted in the absence of any commercial or financial relationships that could be construed as a potential conflict of interest.
